# Implementation of the first adaptive management plan for a European migratory waterbird population: The case of the Svalbard pink-footed goose *Anser brachyrhynchus*

**DOI:** 10.1007/s13280-016-0888-0

**Published:** 2017-02-18

**Authors:** Jesper Madsen, James Henty Williams, Fred A. Johnson, Ingunn M. Tombre, Sergey Dereliev, Eckhart Kuijken

**Affiliations:** 1 Department of Bioscience, Aarhus University, Kalø, Grenåvej 14, 8410 Rønde, Denmark; 2 Wetland and Aquatic Research Center, U.S. Geological Survey, 7920 NW 71 Street, Gainsville, FL 32653 USA; 3 Norwegian Institute for Nature Research, Arctic Ecology Department, The Fram Centre, P.O. Box 6606, N-9296 Tromsø, Norway; 4 UNEP/AEWA Secretariat, African-Eurasian Migratory Waterbird Agreement, UN Campus, Platz Der Vereinten Nationen 1, 53113 Bonn, Germany; 5 Lindeveld 4, 8730 Beernem, Belgium

**Keywords:** Adaptive harvest management, Human–wildlife conflict, Population target, Stakeholder involvement, Structured decision-making, Tundra degradation

## Abstract

An International Species Management Plan for the Svalbard population of the pink-footed goose was adopted under the Agreement on the Conservation of African-Eurasian Migratory Waterbirds in 2012, the first case of adaptive management of a migratory waterbird population in Europe. An international working group (including statutory agencies, NGO representatives and experts) agreed on objectives and actions to maintain the population in favourable conservation status, while accounting for biodiversity, economic and recreational interests. Agreements include setting a population target to reduce agricultural conflicts and avoid tundra degradation, and using hunting in some range states to maintain stable population size. As part of the adaptive management procedures, adjustment to harvest is made annually subject to population status. This has required streamlining of monitoring and assessment activities. Three years after implementation, indicators suggest the attainment of management results. Dialogue, consensus-building and engagement among stakeholders represent the major process achievements.

## Introduction

In Europe and North America, many populations of wild geese are currently burgeoning (Fox et al. 2010; U.S. Fish and Wildlife Service 2015; Fox and Madsen 2017). This follows a combination of protective measures enacted to safeguard populations from overexploitation (Ebbinge 1991), massive land-use changes providing more food for geese (van Eerden et al. 1996; Fox et al. 2005, 2017) and, in some recent cases, climate change on the breeding grounds providing better nesting opportunities (Jensen et al. 2014). Increasing numbers have in some cases led to the degradation of vulnerable tundra vegetation and ecosystem functions (Abraham et al. 2005; Buij et al. 2017). Conflicts with human interests have been exacerbated, for example, due to damage to agricultural crops caused by foraging geese (Bjerke et al. 2013; Tombre et al. 2013) and risks of collisions with aircraft (Bradbeer et al. 2017). Solutions to mitigate the conflict have differed widely between countries depending on the political willingness to pay economic compensation to farmers (van Roomen and Madsen 1992). However, even where compensation or subsidies are provided, conflicts tend to worsen because goose populations continue to increase (Eythórsson et al. 2017; Lefebvre et al. 2017). In response, farmers and airport authorities in some countries have requested that populations be managed to stop the escalation of the conflicts. This has been the case, for instance, with regard to breeding goose populations in Scotland (Bainbridge 2017) and the Netherlands (van der Jeugd 2017), with migratory pink-footed geese *Anser brachyrhynchus* spring-staging in Norway (Direktoratet for Naturforvaltning 1996) and greater snow geese *Chen caerulescens atlantica* in North America (Lefebvre et al. 2017). With regard to resident/sedentary population management, the political decision to cull populations lies with national governments; however, when it comes to migratory species, the issue becomes internationalized. In North America, there is a long tradition for cross-border coordination of management of wildlife populations, both with regard to harvest management (Nichols et al. 2015) and to mitigate conflicts with economic or biological interests (e.g. Lefebvre et al. 2017). In contrast, in Europe there is no such tradition, even though, in principle, there are legislative frameworks in place to achieve this, such as the EU Birds Directive and the Agreement on the Conservation of African-Eurasian Migratory Waterbirds (AEWA). However, in its Strategic Plan 2009–2017 (AEWA 2008), AEWA recognized the need for international coordination and flexible instruments to ensure sustainable use of migratory waterbird populations and to manage populations of waterbirds causing human–wildlife conflicts. The Strategic Plan (Target 2.5) specifically called for international adaptive harvest management plans to be developed for at least two huntable populations.

In this paper, we describe the process leading to the successful implementation and operationalization of the first European adaptive management plan for a migratory waterbird population, namely the population of pink-footed goose breeding in Svalbard and staging/wintering in Norway, Denmark, the Netherlands and Belgium. From the outset, transparency of decision-making and the sharing of knowledge and learning were regarded as essential to the implementation and continued development of the plan. The entire process has been well documented with a record of information relating to the organizational structure, meetings, decisions taken, data, assessments and scientific publications stemming from the process, which are made publically available at the website of the AEWA Pink-footed Goose International Working Group convened to coordinate the implementation of the International Species Management Plan.^1^ Several important lessons have been learned during this process which we think constitutes valuable experiences to enlighten and provide a road map for subsequent processes that are currently in the pipeline for management of other migratory waterbird populations in Europe. The lessons can also be adopted more widely to apply the adaptive management process to populations and nature conservation areas at local, regional and international levels.

## Why an adaptive approach?

North American experts have long advocated for the use of adaptive management of waterbirds in a European context (e.g. Nichols et al. 2007). Some have argued that differences in European conservation policies and cultures, as well as lack of knowledge about the populations, have made such joint initiatives difficult. On the contrary, Nichols et al. (2007) suggest that adaptive management provides a decision-making framework precisely designed for situations where there are sources of difficulty in decision-making, in particular (1) uncertainty about an ecological system and the impact of management actions and (2) potentially conflicting management objectives. Adaptive management uses a formal and structured process to reduce ecological uncertainties through iterative monitoring, adjustment and, hence, learning that improves management over time (Nichols et al. 2007; Williams and Brown 2012). It promotes the participation of stakeholders to agree on clearly defined and measurable objectives, whereby in developing and implementing alternative actions, along with their continued evaluation, stakeholder groups can learn from the process and each other (Reed 2008). By gaining better insights into and understanding of ecological system dynamics, different social values, desired outcomes and potential risks, stakeholders can work towards the collaborative management of challenging situations (Folke et al. 2005). Hence, adaptive management has the potential to embrace different cultural and political viewpoints and values within a democratic and accountable process (Stringer et al. 2006).

In the case of European waterbirds, basic knowledge about population processes governing observed population trajectories is generally poor; harvest data are not collated internationally and, hence, the sustainability of harvest or impacts of other anthropogenic stressors are difficult to evaluate (Madsen et al. 2015a). The AEWA Strategic Plan 2009–2017 promoted the use of adaptive management as a tool to ensure sustainable use of migratory waterbird populations and to manage populations causing human–wildlife conflicts. Under the AEWA Action Plan (AEWA 2015), options for the harvest depend on the status of a given population; populations regarded as vulnerable may only be harvested when an international adaptive management framework is in place. This obliges range states, which wish to continue to hunt such a population, to participate in an internationally coordinated adaptive harvest management initiative. On the one hand, this will require more coordination; on the other hand, it ensures the long-term sustainability of the population as well as continued harvest opportunities. As a corollary, this paves the way for international agreements on well-articulated objectives and improved monitoring for assessing management actions, in particular in relation to the regulation and reporting of waterbird harvests, but also embracing habitat restoration and agricultural damage mitigation measures.

## Initial steps

**Fig. 1:**
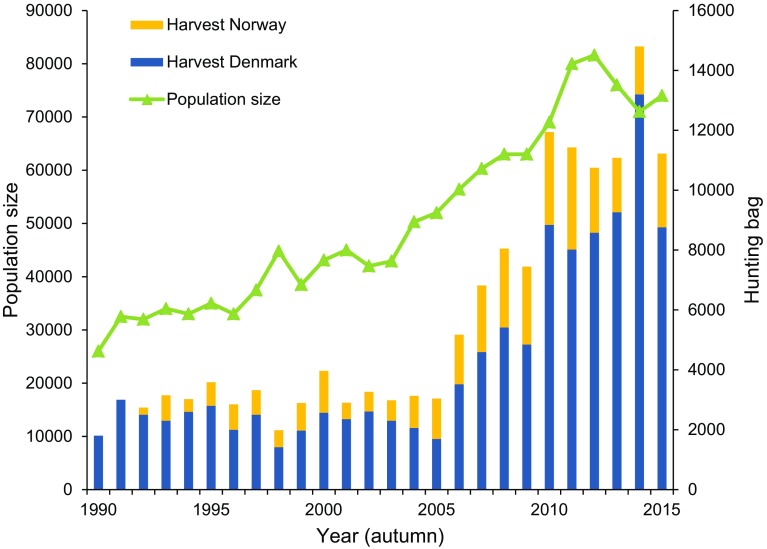
Changes in annual population size and harvest of the Svalbard pink-footed goose, 1989/1990 to 2015/2016 (years on *x*-axis represent autumn). Annual harvest is based on reported bag records in the two countries (starting in Norway in 1992; after Madsen et al. 2016b)

In early 2009, the UNEP/AEWA Secretariat approached the range states of the Svalbard population of the pink-footed goose to establish an adaptive management plan. This population was selected because there was a concrete need for international actions: firstly, because of the intention of the Norwegian government to mitigate an escalating agricultural conflict by stabilizing the population size at the level reached in the mid-1990s, i.e. c. 35 000 geese (Direktoratet for Naturforvaltning 1996); secondly, because of signs of degradation of vulnerable tundra vegetation caused by the geese in Svalbard (Speed et al. 2009), potentially related to its increasing population size (Pedersen et al. 2013); thirdly, the population was regarded as a relatively simple first test case because it involved few range states (Norway, Denmark, the Netherlands and Belgium) with closely related conservation policies; fourthly, it represented a rather well-monitored and studied population with a relatively small but increasing population size (Fig. 1). Authorities from the four key range states responded positively to the initiative although some range states and stakeholders initially expressed their reservations concerning control of population size as a potential action (see below), while peripheral range states (Germany, Sweden) decided not to participate at that point in time. In October 2009, the proposal received support also from the AEWA Technical Committee. Due to its scientific and technical expertise on the ecology and management of the population, Aarhus University was asked to compile the management plan and to act as the coordination unit in the initial phase. To assist, Aarhus University sought advice from experts in the US Geological Survey (USGS), which has long-standing experience with adaptive management of waterbirds (Johnson et al. 2015a). This led to a formalized technical collaboration between the two institutions which has been in operation since.

**Fig. 2:**
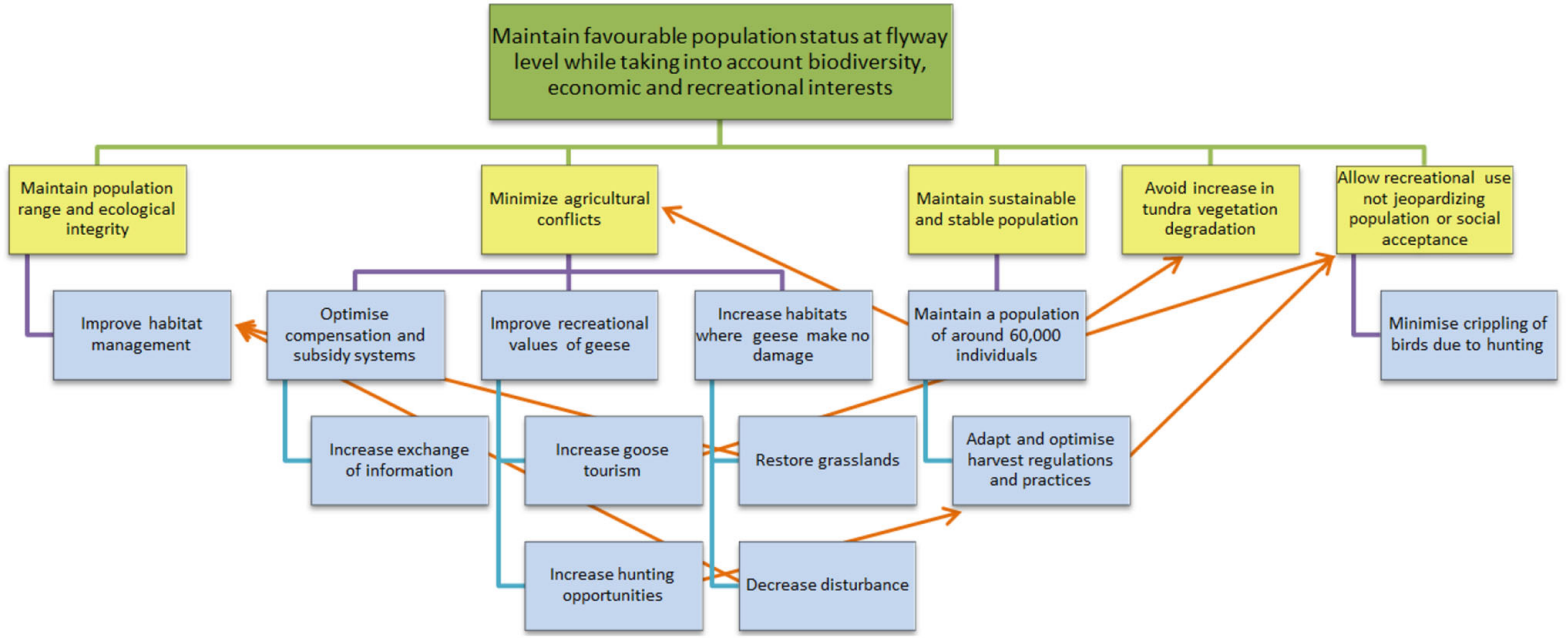
Hierarchy of objectives for the International Species Management Plan for the pink-footed goose. *Top level* goal (or strategic objective); *second level* fundamental objectives (which are supposed to be SMART, i.e. Specific, Measurable, Achievable, Results-oriented and Time-fixed); *lower levels* means objectives (or alternative key actions) to reach the fundamental objectives. *Red arrows* show positive feedbacks between objectives

Initiated by the UNEP/AEWA Secretariat and hosted by the Danish Nature Agency, an international workshop was held in Denmark in November 2010 to bring together key stakeholders, solicit their input and start the planning process. Authorities from the four range states, international NGOs (Wetlands International, BirdLife International, European Federation of Associations for Hunting and Conservation) and experts were invited. The authorities heading the national delegations were also invited to bring representatives from relevant national NGOs, including representatives from farmers’, hunters’, ornithological and nature conservation organizations. At the workshop, a proposed management plan was presented, which outlined a biological assessment of the population status and management issues. At the meeting, participants were introduced to the theory, principles and procedures of adaptive management. Based on an analysis of management issues, pathways and root causes, participants discussed and drafted overall goal, objectives and key actions to achieve the objectives (here reformulated in terms of an objectives hierarchy in Fig. 2). Based on the outcomes of the initial workshop, a draft International Species Management Plan (ISMP) was compiled, which detailed proposals for a framework of action according to the principles of adaptive management, with clearly defined objectives and key actions as well as proposed milestones and an organizational structure. The draft went through a review process including commenting by experts, range states’ authorities as well as the AEWA Technical and Standing Committees. In March 2012, a final draft was submitted for the 5th Meeting of the Parties to AEWA, and in May 2012, the Meeting of the Parties unanimously adopted the plan (Madsen and Williams 2012).

## Population target setting

One of the most challenging issues was to reach agreement on population management as a means to avoid further escalation of agricultural conflicts with geese as a result of crop damage and further possible degradation of tundra vegetation. Even though management and regulation of numbers of wildlife species is widely used as a conservation management tool in Europe at a national scale, e.g. to manage populations of deer, large carnivores, foxes, crows and cormorants, there is no such precedence of regulation at an international level (Williams and Madsen 2013). Hence, principles for population target setting at the international level did not exist, except for defining minimum viable population sizes for endangered populations (Sanderson 2006). Initial stochastic modelling predicted that the population could be sustained at a level of c. 40 000 (the population size in 2002) and that it would be relatively robust to increases in harvest levels (Trinder and Madsen 2008). This population level was also close to that which the Norwegian management plan had proposed as a population target (Direktoratet for Naturforvaltning 1996). In general, some representatives from the statutory agencies and NGOs from the Netherlands and Belgium expressed concerns regarding the status of the pink-footed goose as a conservation flagship and an asset in their countries. However, they were not in favour of ‘hundreds of thousands’ of geese because of the predicted damage to agriculture and consequent cost to society from payment of compensation to farmers (money which might be taken from nature conservation budgets). Furthermore, the principle of managing this relatively small population with negligible agricultural impact in these countries raised ethical questions because conflicts with far more numerous species (such as greater white-fronted geese *Anser albifrons* causing serious conflicts with agriculture and damage payments, especially in the Netherlands) were not addressed. Representatives from BirdLife International expressed concern regarding defining a ceiling for population size, favouing ‘natural’ population growth. Others, primarily from hunters’ organizations, preferred more geese in order to gain shooting opportunities, but they also understood the concerns raised by the farming community. The Norwegian delegation, on the other hand, reiterated that a continued growth of the population was unacceptable due to the agricultural damage and the risk to the Svalbard tundra and, if a population ceiling could not be agreed, Norway would take their own initiatives in an attempt to control the population. Finally, an agreement was reached to propose a population target around 60 000 individuals (spring population size) at the first international workshop where open, frank and constructive face-to-face discussions enabled all participants to state their positions, yet still come to a consensus. From a population conservation point of view, this level was regarded as relatively safe to prevent risks of overexploitation and population collapse, provided that an adaptive harvest management system, including close monitoring, was put in place. BirdLife International agreed to the target in this specific case in order to allow for a test case. The Dutch and Belgian delegations accepted the target, provided that it could be re-negotiated when new knowledge was made available to demonstrate that it was justified, i.e. that the population size actually mattered in relation to the amount of crop damage and tundra degradation. The Norwegian delegation accepted the target with the notion that this was to be regarded as a maximum (upper target).

These perspectives also manifested themselves in the ‘utility’ or acceptance of variance around the population target, e.g. population sizes between about 50 000 and 70 000 were acceptable (and thus have high utility), while those outside this range were undesirable (and thus have low utility) (Johnson and Madsen 2015). All parties agreed that the population target would be open for review and re-negotiation, based on scientific evaluation and consultation with stakeholders as stipulated in the ISMP. Any change was envisaged to occur by the time of the revision of the plan, i.e. in 2022, but if circumstances indicated that a revision was necessary before, this was not ruled out. In other words, the agreed population target was a ‘social construct’ (Williams and Madsen 2013) based on a combination of biological evidence, beliefs and values. One can argue that this represents a state of ‘least mutual dissatisfaction’ among the stakeholder interests (I. Bainbridge, pers. comm.).

It was also proposed that the population target should be reached and maintained by means of optimizing hunting regulations and practices in the range states where hunting is currently allowed (Denmark and Norway). Hence, the Dutch and Belgian delegations abstained from considering opening a hunting season or allowing derogation shooting.

## Implementation phase

In August 2012, an ISMP implementation workshop was held in Longyearbyen, Svalbard, hosted by the Norwegian Environment Agency. The meeting was convened to discuss and agree on the organizational and procedural requirements needed to implement the ISMP to achieve its objectives. Each of the fundamental objectives was discussed in order to agree on monitoring protocols and follow-up actions; however, focus was on the implementation of an adaptive harvest management strategy, proposed to be launched from the 2013 hunting season onwards. At that time, there were uncertainties in population dynamics and thus how the population would be impacted by increasing harvest, combined with uncertainties of the effectiveness of hunting regulations to adjust harvest levels. Therefore, scientists argued that it would be optimal to use a model with 1-year decision-making and adjustment of harvest regulations. The Norwegian and Danish delegations as well as hunting organizations advocated for a 3-year decision-making cycle in order to maintain stable hunting regulations which would be easier to administer (and consistent with existing decision-making cycles in wildlife harvest regulations in Denmark and Norway) and were thought to be more acceptable by the hunting communities. Furthermore, the perspective of launching a harvest strategy with the aim to reduce the current population size raised some concerns about social acceptability, particularly articulated by the Dutch and Belgian delegates; they advocated for a gradual population reduction rather than a massive sudden reduction which might be adversely received by the public. To avoid an unforeseen negative impact of either the harvest or adverse environmental conditions, it was also proposed to introduce an option for closing the hunting season for one year (an emergency closure). It was agreed that Denmark and Norway would aim to implement a regulated system of hunting starting in 2013, based on a 3-year cycle of adaptive harvest management, with annual reviews to carefully assess population dynamics and other environmental factors to ensure the sustainability of hunting and the favourable status of the pink-footed goose population.

Subsequently, a model framework (see Box 1) was developed to predict an optimal harvest strategy for a 3-year period (2013–2015), providing annual updates to evaluate the need for emergency closures. The work was subject to an independent peer review, requested by the Dutch delegation. Furthermore, the description of the models, the use of demographic data and environmental variables (e.g. use of spring weather conditions in Svalbard as a predictor of the production of young) have been published in peer-review journals (Jensen et al. 2014; Johnson et al. 2014a). The open publication of decisions taken, annual assessments (Population Status and Adaptive Harvest Management reports) and other related scientific work was instilled in the implementation phase, in particular with the development of the ISMP website as a means for disseminating information. From the start, there was a clear desire among the parties to have a transparent and reviewable decision-making process and scientific knowledge base that was open to scrutiny and that would foster continued learning and development based on feedback not only from within the working group but also from outside.

**Box 1:** Procedures of annual adaptive harvest management of the pink-footed goose population in a nutshell

The development of an adaptive harvest management (AHM) strategy requires the specification of four elements: (a) a set of alternative population models, which bound the uncertainty about effects of harvest and other environmental factors, (b) a set of probabilities describing the relative credibility of the alternative models, (c) a set of alternative harvest quotas from which to choose and (d) an objective function, by which alternative harvest strategies can be evaluated. An optimal management strategy prescribes a harvest quota for each and every possible set of model probabilities, and for population abundance and environmental conditions that may be observed at the time a decision is made
Nine models of pink-footed goose dynamics describe competing hypotheses about how reproductive and survival rates might vary over time. The models focus on whether spring temperature and density dependence influence survival and/or reproduction. Bayesian probabilities are used to express the relative ability of each model to accurately predict the changes in population size that actually occur, and they are updated each year using monitoring information. In the figure below are the time sequences of the aggregate probabilities on models that incorporate (A) density-dependent survival, (B) density-dependent reproduction and (C) days above freezing in May in Svalbard in the reproductive and survival processes

The four elements of AHM (models, model probabilities, alternative quotas and objective function) are used each year to calculate an optimal harvest strategy designed to maintain the population near the goal of 60 000. The optimal harvest strategy is a large lookup table that is difficult to display graphically. Below is a simplified representation of the strategy for model probabilities in 2016, in which a series of yes–no questions are asked (yes is the left branch; no is the right branch) about the abundance of adults and young (A and Y in thousands, respectively) and the number of days above freezing in May in Svalbard (DAYS). The approximate harvest quota (in thousands, to the nearest 2.5) is given at the ends of the branches

## Adaptive management launched

### Organizational structure

Subsequent to the implementation workshop, the range states agreed on the establishment of an International Working Group (IWG) to review and guide the implementation of the ISMP. The composition of the IWG is similar to the original group that met to develop the ISMP and later on to launch its implementation. Range states were also encouraged to establish national working groups to implement and review implementation of decisions at national levels and to provide feedback and input to the IWG. The IWG’s mandate is to make recommendations that are to be implemented nationally in each country. For example, in Denmark, recommendations on hunting regulations will be passed on to the national Wildlife Management Council (composed of national NGOs, advisory to the Minister of Environment and Food), which will recommend a decision to be signed by the minister. To coordinate the ISMP monitoring and assessment work, as well as organizing IWG meetings and facilitating internal and external communication, a coordination unit has been established at Aarhus University under a Memorandum of Understanding with the UNEP/AEWA Secretariat. The unit works closely with the UNEP/AEWA Secretariat, and an agreement between Aarhus University and USGS ensures a tight collaboration on the technical development of adaptive management, knowledge transfer and annual updating of adaptive harvest management assessments.

The IWG met for the first time in April 2013, to launch the implementation of adaptive harvest management as well as habitat-related issues of the ISMP. Since then, the IWG has met annually. The number of participants has increased from around 20 in 2010–2013 to 30 in recent years. Participants at the meetings have represented a wide array of stakeholder groups, e.g. statutory agencies, hunting, farming and conservation organizations not only at international and national levels, but also at regional and local levels. The involvement of regional and local representatives enabled direct dialogue between multiple levels of governance and management and, furthermore, has provided a means of dissemination and advocacy for IWG activities as well as a source of learning at grass-roots levels. Focus at the meetings has been on taking collaborative actions at multiple levels to fulfil the fundamental objectives of the ISMP, as well as setting up appropriate monitoring activities and collectively assessing the first outcomes of actions.

### Monitoring

The IWG has recommended monitoring activities and protocols to assess the status of the ecological system and the progress of the ISMP actions in fulfilling each of the objectives (Fig. 2). The monitoring has been funded by statutory agencies in the four range states.

**Fig. 3:**
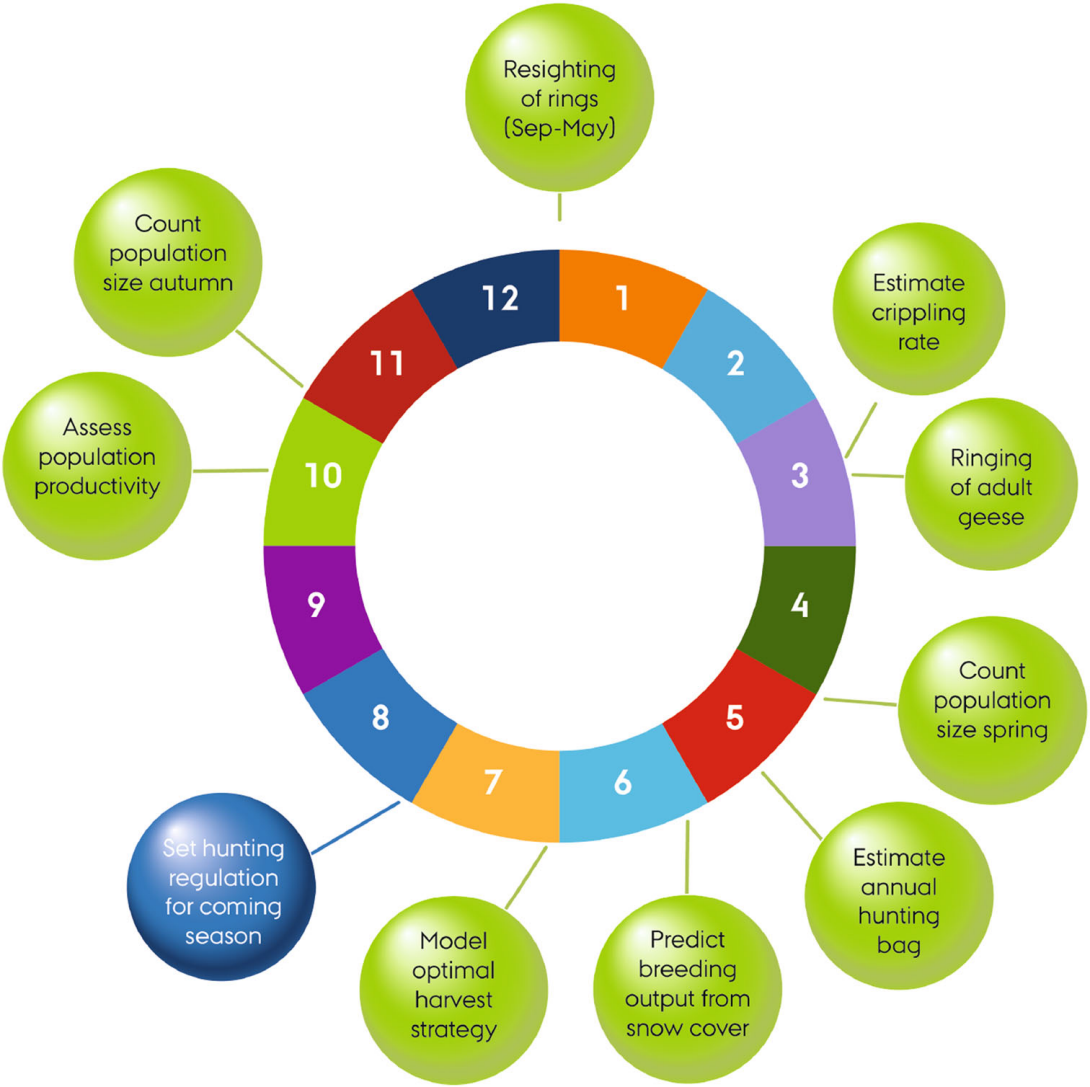
Annual cycle in monitoring, assessment and decision-making in the adaptive harvest management of the pink-footed goose

The primary focus of the monitoring programme has been to ensure that the population is maintained at a favourable and stable level by adaptive harvest management procedures (Box 1). The most important monitoring variables have been population size, demographic parameters (fecundity, survival), spring weather conditions in Svalbard as a proxy for production of young, as well as harvest in Denmark and Norway. These activities are spread over the annual cycle (Fig. 3). Until recently, population estimates were based on synchronized ground surveys of geese in the range states in early November (in some years also late December), but their timing in mid-hunting season is rather unfortunate. As a consequence, these surveys have been supplemented by a synchronized survey in early May, i.e. shortly before the geese migrate to the breeding grounds. The harvest of geese in Norway and Denmark is reported online by hunters, and by mid-April, a reliable estimate of the harvest in the preceding hunting season can be provided. On 1 June each year, the weather conditions in Svalbard are assessed based on temperature records from selected meteorological stations.

To monitor levels of agricultural conflicts, the IWG has recommended the standardized monitoring of the costs of goose damage (in terms of compensation, subsidies paid and complaints) and the associated administration costs. This will enable the IWG to determine the plan’s effectiveness in alleviating these conflicts and assess if there is a relationship between the population size and actual economic costs related to crop damage, i.e. to evaluate the justification for the population target. As a matter of concern, it has also been recommended to monitor the extent and intensity of goose-caused degradation of the tundra vegetation. So far, this is carried out in the Isfjorden area in Svalbard, using a course grid of transects which are monitored at intervals of 3–4 years and a finer grid of transects in one area which is monitored at intervals of 1–2 years (Pedersen et al. 2013; Anderson et al. 2016). To monitor the social acceptability of hunting, which primarily relates to the ethical issue of potential risk for crippling birds by shotgun shooting, the IWG has recommended that the rate of crippling is monitored at regular intervals as a proxy measure. Finally, the range and ecological integrity of the population are monitored by the systematic surveys of the distribution of geese, their habitat use and efforts taken to restore habitats and reduce disturbance.

### Harvest management actions

**Fig. 4:**
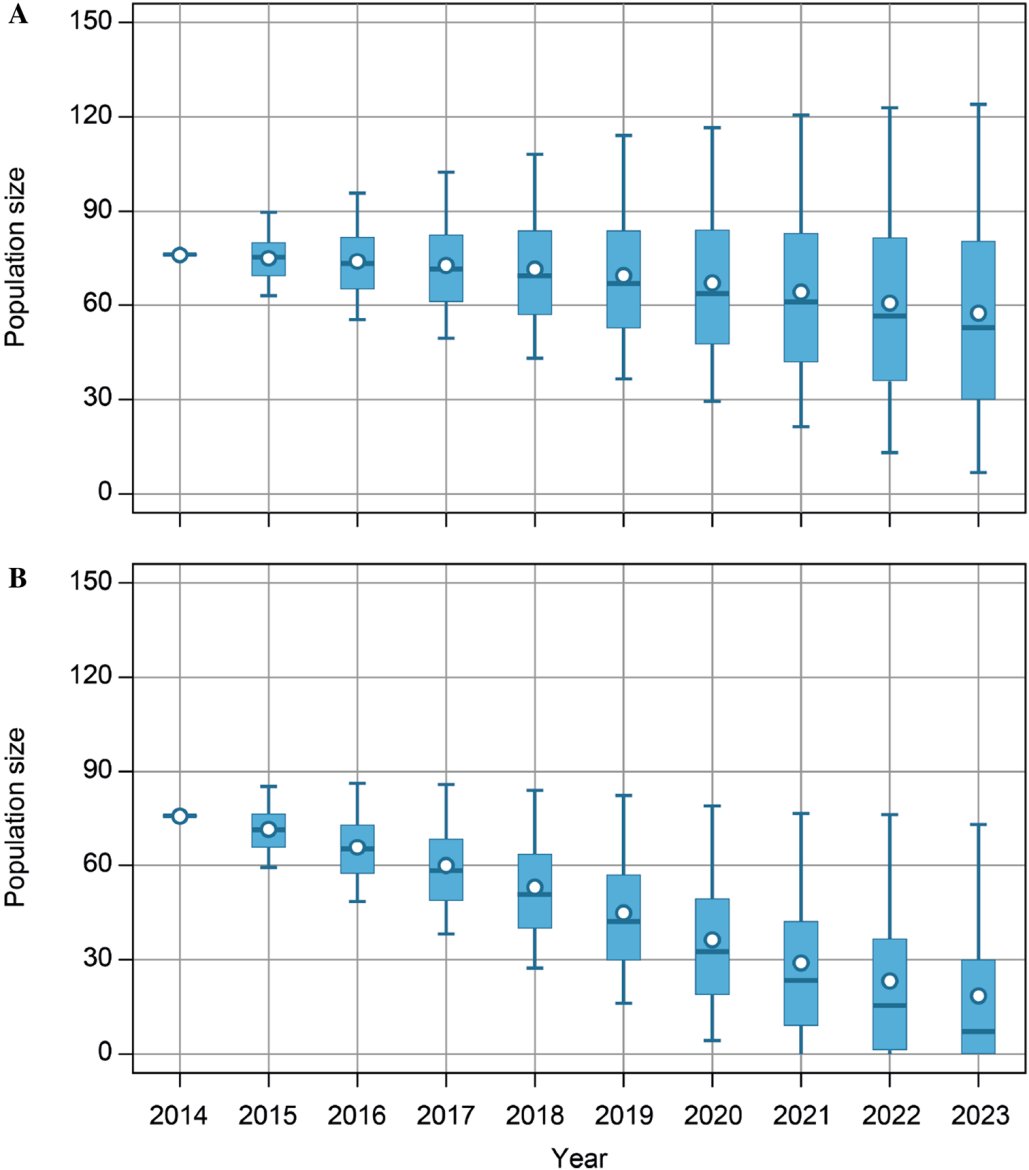
Projection of pink-footed goose population size (in thousands) based on current model weights and assuming a harvest of 11 300 (average of 2011–2013) (**a**) and 15 000 (**b**). *Vertical lines* represent 95% confidence limits, *boxes* are the interquartile ranges, *horizontal lines* are medians, and the *open circle characters* represent the means. Projections of population size were based on observed, post-harvest population size in 2013, random variation in positive temperature days in Svalbard in May (as a proxy of advancement of spring) and model process error. Each time series was simulated five thousand times (after Johnson et al. 2014b)

In spring 2013, the population size was estimated at an unprecedented peak of 81 600 individuals (Fig. 1) and the annual total harvest was 11 300 individuals (averaged over the 2011–2013 hunting seasons). Hence, to reduce the population to the 60 000 target it was deemed necessary to increase the pink-footed goose harvest. The optimal annual harvest for 2013–2015 was 15 000 individuals. Population modelling predicted that if the present total harvest was maintained, the population would reach the target within 8–9 years; if the harvest level was increased to 15 000, the target would be reached after 3 years (Fig. 4). The IWG recommended that the higher harvest should be pursued in collaboration between Norway and Denmark where hunting was permitted.

Using hunting as a management tool has not been applied in an international context in Europe. The outcome of any harvest regime will depend on the effectiveness of the regulatory tools at hand as well as the interest and acceptance by hunters to shoot more or fewer geese. In the case of the pink-footed goose, the required increase in harvest could be achieved in various ways, including better local organization and increasing the effectiveness of goose hunting, as well as lengthening open seasons. All these options were discussed in the IWG. In both Norway and Denmark, research projects have been designed to make field tests of how local organization of goose shooting can contribute to the achievement of ISMP objectives. These experiments have been based on voluntary agreements between landowners and hunters to optimize and organize shooting in time and space within the hunting season. The first results indicated that intermittent hunting activities and better organization with fewer and more widely distributed teams of hunters using decoys to attract geese can lead to increased harvest and at the same time reduce disturbance and, probably, reduce wounding (Jensen et al. 2016a, b, 2017; Madsen unpubl. data). Hence, while local organization can be used to increase the harvest, it remains to be demonstrated how this can be used to regulate the harvest once the population approaches the agreed target and harvest has to be reduced. However, in Norway, a quota system for a national harvest is currently being tested, and the spatial and temporal harvest distribution can potentially be controlled by local networks of landowner–hunter groups.

Season length regulation is a management option widely used in Europe, although the effectiveness of changing hunting season lengths to adjust harvest levels is debated (Sunde and Asferg 2014). In response to the IWG recommendation for increased pink-footed goose harvests, the Danish Wildlife Management Council recommended that the open season be extended from September to December to include January commencing with the 2014/2015 hunting season. In Norway, the potential hunting season is shorter because the geese arrive from Svalbard in mid-September and normally abandon the staging areas in the start of November due to snowfall (Jensen et al. 2016a); therefore, extending the season in Norway was not adopted as an appropriate action. The outcome of the extension of the hunting season in Denmark was that the Danish hunting bag increased by 35% compared to the average of the previous three seasons; in the hunting season 2014/15, 47% of the total harvest was taken in January (Madsen et al. 2016a). The combined Danish and Norwegian harvest totalled 14 800 geese (Fig. 1), i.e. close to the 15 000 optimum. Hence, first impressions suggested that hunting season extensions can be a powerful tool; however, it has to be tested more frequently (which will take several years of iterations of the adaptive cycle) to examine the combined effects of winter weather conditions and goose distribution on the harvest. Furthermore, the effect will also be dependent on hunters’ ability, and willingness, to adjust their activity. In this context, it is recognized that there is a need to better understand hunter motivations and values and, thus, their likely behavioural responses to management actions and regulatory changes. Such sociological studies are currently underway in both Norway and Denmark. Early indications from these studies suggest the need for targeted training of potential and existing hunters, raising awareness of effective and efficient goose hunting methods that maximize opportunities whilst minimizing disturbance (Williams unpubl. data).

Once the population approaches the target, it will be necessary to coordinate the annual harvest between Norway and Denmark to share the annual ‘quota’. The IWG, supported by national working groups, has recommended that the total harvest should be shared according to harvest levels for previous hunting seasons in each country, i.e. 30% to Norway and 70% to Denmark. It still has to be demonstrated how this shall be controlled and traded, but the proposal to introduce a Norwegian quota system will be the first step in the right direction.

The ISMP promotes sustainable hunting and implicitly recognizes the need for maintaining the social acceptability of hunting, when used as a management tool. A much debated issue, particularly in Denmark and reflected in concerns raised within the IWG, has been the wounding of birds due to shotgun shooting. In the early 1990s, 36% of live-caught X-rayed adult pink-footed geese carried shotgun pellets in their tissue (Madsen and Noer 1996); this led to a Danish action plan to reduce the wounding of geese based on targeted campaigns to train and make goose hunters aware of the importance of shooting at close ranges and using appropriate ammunition. The campaigns resulted in a decrease in rates of geese carrying shotgun pellets, monitored by regular X-raying (Noer et al. 2007). In 2009 and 2011, an increase in rates of geese carrying shotgun pellets was observed. In response, the IWG recommended that it was preferable to see a continued decline in these rates rather than setting a fixed target as an ISMP objective. To follow up previous monitoring, in spring 2015 and 2016, X-raying of captured birds was conducted as part of an ongoing programme to cannon-net and mark geese in mid Norway. In addition, new awareness campaigns focusing on the avoidance of wounding have been initiated in both Norway and Denmark. The first results appear to be positive in terms of a subsequent decline in rates of geese carrying shotgun pellets (Holm et al. 2015); however, because of the longevity of geese, it will take years to see a major effect in the segment of older birds.

Harvest management actions of the ISMP have been focused at the international and national levels but the research and management projects mentioned above, instigated in connection with the ISMP, endeavour to identify ways to bridge the gap between national regulatory actions and local management actions. Participation of stakeholders, in this case hunter representatives from local to international levels, in various ways within the ISMP process, e.g. by attending IWG meetings and participating in collaborative research projects, has enabled their local knowledge to be expressed and influenced the development of beneficial actions for all concerned.

### Agricultural conflict management actions

The ISMP aims to reduce agricultural conflicts, partly by stabilizing the population size and partly by optimizing existing practices to mitigate crop damage by exchanging experiences with agricultural management initiatives. Data made available from the Norwegian authorities on the annual amount of subsidies suggest that the size of the population scales with the costs of agricultural conflict management in Norway (e.g. Eythórsson et al. 2017), and this is backed by scenarios produced by a simulation model (Baveco et al. 2017). Such information can be coupled to explore the relative cost effectiveness of various practices.

### Habitat restoration actions

Historically, pink-footed geese foraged on semi-natural grasslands in their key staging and wintering areas in north Norway, Denmark, Friesland in the Netherlands and Flanders in Belgium (Madsen 1984; Kuijken et al. 2006; Kuijken 2010; Tombre et al. 2010). Some of these areas have been protected (e.g. under the EU Bird and Habitat Directives), but gradually many grasslands with formerly high nature values have become cultivated. Some of the remaining semi-natural grasslands have declined in quality, partly due to lack of livestock grazing and resulting in a rank sward such as that occurred in parts of the northern staging areas in Norway (Tombre et al. 2010). In some regions, new crops have been introduced in former grassland areas, such as maize and potatoes, which are quickly discovered and exploited by geese (Cottaar 2009; Kuijken and Verscheure 2016). As a consequence of the combination of loss of quality and quantity of the traditional habitats and introduction of new crops which provide geese with high-energy food (Fox and Abraham 2017), geese tend to move to intensively cultivated grasslands or arable land which is fuelling the agricultural conflict. The ISMP promotes actions to restore beneficial habitats in key feeding areas, as well as to reduce disturbance at sites where they do not cause damage to farmland crops, e.g. when foraging on waste grain in stubble fields in autumn. An EU LIFE project, supporting ISMP habitat objectives, is currently underway in Flanders, Belgium, to restore 200–300 ha of semi-natural grassland, which is beneficial not only for pink-footed geese but also for wider biodiversity and nature conservation objectives (Kuijken and Verscheure 2016).

### Annual assessment process

As recommended, the optimal harvest has been updated annually. The process has been designed around key decision milestones within the national agencies in Denmark and Norway and a timeline was planned to allow for an administrative emergency closure prior to the opening of the hunting season. This means that (1) the monitoring data have to be compiled and quality assured (by the experts in the IWG) in early June each year, i.e. one month after the spring survey, (2) the updates of the models have to be made and quality assured (by the IWG) in June, (3) the assessment has to be presented to the national decision makers in June/July, and (4) the hunting regulations must be set and communicated to the hunting community in August (Fig. 3). The Danish Wildlife Council and the National Nature Agency asked for an even faster process, and from 2015 onwards, the process has therefore been compressed to enable regulatory decisions to be taken in the second half of June.^2^

### Stumbling and adjusting

In spring 2015, the population survey found a total of 59 000 geese. This was much lower than predicted based on the optimal harvest strategy, despite the fact that record-high 14 800 geese had been harvested. According to the annual update of the harvest strategy performed in June 2015, this would result in the population dropping below the 60 000 target, if the 3-year harvest strategy was maintained for the season 2015/16. Based on the 3-year harvest strategy agreement, this situation required an emergency closure of the season. However, the Danish Wildlife Council discussed an alternative, namely to use the predictions based on a 1-year strategy. According to this, a total of 6700 pink-footed geese could be shot in the coming season without drawing the population below the 60 000 target (i.e. c. 2000 to Norway and 4700 to Denmark). The Danish and Norwegian authorities agreed to use this option. Continuing with a 3-year harvest strategy, including options for emergency closures, could lead to undesirable swings between fully open and fully closed seasons in the future. It was also difficult to justify why an emergency closure was necessary, given that harvest assessments based on a 1-year strategy suggested that there were still opportunities for hunting, albeit at reduced levels. Consequently, due to the uncertainties about the population response to increased harvest, the Danish Wildlife Management Council recommended that the ISMP should deploy a 1-year harvest strategy. These arguments were subsequently accepted by the four range states and the IWG. To reduce the harvest in the 2015/16 hunting season, the Danish Wildlife Management Council recommended that the January extension be withdrawn. In Norway, there was not sufficient time to adjust regulations, but hunters were asked to reduce their harvest of pink-footed geese to stay within the quota of 2000 birds.

When the population survey was carried out in November 2015, a total of 74 800 geese were counted. This highlighted the fact that the 59 000 counted in spring 2015 was an underestimation (i.e. missing of the order of c. 11 000–12 000 geese). The authorities in Denmark and Norway were informed of the revised population data but the Danish Wildlife Management Council recommended maintaining the decision to close hunting in January 2016. This was seen as an important principle of the adaptive harvest management regime, whereby decisions made based on the best available data at the time are adhered to. Nevertheless, scientific experts within the IWG were asked for ways to avoid such biases and sensitivities in the future monitoring and assessments. In spring 2016, extra efforts to search for goose flocks outside the known range were made; it turned out that large flocks of geese had started to utilize new sites in mid Norway and, most surprisingly, that a group of c. 3000 geese had started to migrate via Sweden to spring-staging areas in western Finland (see Madsen et al. 2016b). Despite the fact that pink-footed geese are known to be highly site-faithful to wintering sites in Belgium and the Netherlands, rapid and unforeseen shifts in staging and wintering distributions have been observed at an accelerating rate (e.g. Madsen et al. 2015b). These changes demonstrate that the ground surveys (so-called ‘total counts’ assuming that all flocks are found) are likely to result in underestimates of the true population size, but with an unknown source of bias from year to year. An alternative to ground surveys has been developed based on capture–mark–resightings (CMR) of neck-banded individuals (Ganter and Madsen 2001). Basically, the population size can be estimated if the number of live neck-banded birds in the population can be estimated and the ratio of marked versus unmarked individuals is registered. This earlier analysis has now been updated, suggesting that the ground surveys generally result in a slight and consistent underestimation of the population size and, more specifically, that the spring 2015 estimate was biased low (Clausen et al. unpubl. data). Furthermore, the CMR methodology has proved to be effective and can be used to check the annual population estimates in a timely manner to meet the June decision-making deadline.

In June 2016, the annual updates predicted that there were opportunities to shoot more geese in the coming hunting season to realize the agreed population target. This assessment resulted in a decision to re-open hunting of pink-footed geese in January 2017 in Denmark and that there was no need for setting a quota on the harvest in Norway. The ground survey in spring 2015 demonstrated that the adaptive harvest management process is highly sensitive to bias in a single number. This sensitivity is amplified by the fact that there seems to be little density dependence affecting the population development at the present (Box 1; Johnson and Madsen 2016). When the population size approaches the population target, this results in a knife-edge decision-making process that alternates between ample hunting opportunities and a season closure. Stumbling over the survey problem in this early phase has had a short-term adverse effect on the credibility of the process (manifest in negative voices on social media and in hunters’ magazines). However, the structured and rigorous process of the ISMP adaptive management system captured this deficiency and adjusted accordingly by sharpening and extending the monitoring methodology. Furthermore, in the long term, this episode may strengthen the ISMP process by highlighting continued learning and adjustment.

Despite the fact that the number of geese harvested in the 2015/2016 hunting season was not sufficient to reduce the population size as recommended by the IWG, the population appears to have started to decline (Fig. 1), which is in accordance with the predictions (Fig. 4). The coming years will demonstrate if the harvest rate, and the regulatory tools at hand, will be sufficient to stabilize the population around the target and if this is resulting in reduced damage to agriculture and vulnerable tundra vegetation.

## Conclusions and perspectives

The implementation of the ISMP for pink-footed geese has been viewed, by some, as a relatively resource-demanding process. From the onset, it was regarded as essential to have regular (annual) meetings in the IWG (as well as national working groups), setting up a dedicated coordination unit and a technical assessment procedure. The high frequency of meetings and high level of communication have been instrumental in maintaining momentum and forming the process. We think it is fair to say that when AEWA launched its Strategic Plan 2009–2017, very few managers and scientists involved in waterbird management in the region knew much about the practice of adaptive management. Hence, the ISMP process for the pink-footed goose has entailed substantial technical and institutional learning under the guidance of US experts. Participants in the IWG have gradually become familiar with adaptive management (which has changed mind-sets), but it also became obvious that the end users (managers, hunting and conservation organizations) also needed to go through a learning process, having initially had little understanding of the concepts. This has led to a series of misunderstandings, and some groups have used the situation to attempt discrediting the use of adaptive management (particularly on social media in Denmark), also observed in other sectors (Walters 2007). The lessons learned are that there is a need for widening the understanding of adaptive management in order to take full advantage of the framework. Thus, there is a need for capacity building at undergraduate, graduate and professional levels (Johnson et al. 2015b), and since 2015, Aarhus University has started providing courses at all three levels. However, the best way for newcomers to learn is probably to get involved in case studies involving adaptive management.

The ISMP for pink-footed geese has been regarded as a ‘low-hanging fruit’ because of the relatively few countries involved, relatively common conservation policies and cultures, as well as good biological knowledge. Undoubtedly, this created a good starting point, and it has made it easier to discuss and reach agreements on difficult issues such as population target setting and the use of hunting as a management tool. However, as described above, there are political differences between countries and stakeholders, and surprises keep emerging in the ecological system. However, the structured process and the annual reviews have facilitated social and technical learning, supplemented by open publication of all IWG documents that has facilitated trust in the process within and outside the group. Furthermore, the very participatory nature and structure of the IWG with face-to-face discussions has enhanced trust-building amongst participants.

The IWG, including observers, has grown over the years and there has been a high degree of continuity in the people involved, which can be seen to reflect the belief that the process has been worthwhile and results-oriented. Concrete advances have been made in terms of evidence-based management actions, but we believe that participants also appreciate the joint learning process, and this applies to all groups involved, including managers, NGOs and scientists, with some funding their own attendance. While some stakeholders have primarily been involved in order to learn and prepare for a wider application of the concept, others have proactively taken advantage to use the arena to achieve influence and translate recommendations into action. This also includes the scientists who have received funding to carry out targeted research to build models, evaluate efficiency of various harvest regulation tools, sociological studies and develop monitoring systems. It has taken time to reach a common understanding of the different roles and responsibilities of participants in the process, from the international to the local levels, and this will remain an important issue to address because there will be an exchange of participants and new stakeholders may become involved.

Despite some detours along the road, we now have a ‘proof of concept’, where feasible, quantifiable and measurable indicators for all ISMP objectives have been pursued and monitoring activities reflect the focus on continued evaluation and progress towards these objectives as part of an adaptive management process. There is now a forum in place to deal with emerging issues and provide quick responses to unexpected events. We have also seen that the plan process has been a stimulus and encouragement for habitat restoration actions. There continues to be much learning to be done regarding the technical issues of adaptive harvest management. Further, there will also be a need to gather more information about the effects of population size on agricultural damage and tundra degradation. The participatory nature of the adaptive management process has facilitated notable steps forward in several ways: the inclusion of representatives spanning international to local levels, the integration of scientific and local knowledge as well as acceptance of accountability (e.g. scientists accountable to provide best available data to inform decisions and hunters adhering to harvest quotas and variable hunting seasons) are all seen as beneficial in the management of complex socio-ecological systems (Berghöfer et al. 2008). Growing awareness within the IWG of widely varying social values has also focused attention on different priorities and choices, many requiring trade-offs, as well as attitudes towards risk and regulatory changes, particularly in relation to population target setting. Our experiences mirror many of the lessons learned in the US in managing waterfowl harvest (Johnson et al. 2015a).

Based on the preliminary results of the ISMP for the pink-footed goose, the 6th Meeting of the Parties to AEWA (November 2015) endorsed the development of a broader multispecies management platform for geese in Europe. The intention is to establish an organizational structure with an international working group responsible for decision-making and a data centre to provide monitoring data and assessments based on input from the participating countries. The species to be included in the first phase, besides the pink-footed goose, are the taiga bean goose *Anser f. fabalis*, the northwest European population of the greylag goose *Anser anser*, and the barnacle goose *Branta leucopsis* (three populations). This increases not only the number of countries involved but also the diversity in conservation policies, cultures, complexity in management and conservation issues. Furthermore, for some of the species the knowledge about population dynamics is fragmentary or complex. The increased complexity will require patience in the negotiations and planning phases, and due to the fact that many participants will be new to the adaptive management processes, an educational phase should be incorporated. With such a multispecies programme unfolding, the workflow will become more formalized. From the ISMP for the pink-footed goose, it has been apparent that the progress has been driven by a highly motivated core group. In a larger setting, there is a risk that this drive will be diminished, and it will be important to give space to establish focused sub-groups for building trust and to generate momentum in the process.

## Footnotes

1 See http://pinkfootedgoose.aewa.info.

2 See annual population data and harvest assessments at http://pinkfootedgoose.aewa.info/report_series.
